# Pre-surgical Caregiver Burden and Anxiety Are Associated with Post-Surgery Cortisol over the Day in Caregivers of Coronary Artery Bypass Graft Surgery Patients

**DOI:** 10.1007/s12529-019-09775-6

**Published:** 2019-02-21

**Authors:** Claudio Singh Solorzano, Andrew Steptoe, Elizabeth Leigh, Tara Kidd, Marjan Jahangiri, Lydia Poole

**Affiliations:** 1 Department of Behavioural Science and Health, University College London, 1-19 Torrington Place, London, WC1E 6BT UK; 2 School of Natural Sciences and Psychology, Liverpool John Moores University, Tom Reilly Building, Byrom Street, Liverpool, L3 3AF UK; 3 Department of Cardiac Surgery, St George’s Hospital, University of London, Blackshaw Road, London, SW17 0QT UK

## Abstract

**Background:**

The relationship between pre-surgical distress and diurnal cortisol following surgery has not been investigated prospectively in caregivers of coronary artery bypass graft (CABG) patients before. We aimed to examine the relationship between pre-surgical anxiety and caregiver burden and diurnal cortisol measured 2 months after the surgery in the caregivers of CABG patients.

**Method:**

We used a sample of 103 caregivers of elective CABG patients that were assessed 28.86 days before and 60.94 days after patients’ surgery. Anxiety and caregiver burden were assessed using the anxiety subscale of the Hospital Anxiety and Depression Scale and the Oberst Burden Scale respectively. Saliva samples were collected to measure cortisol area under the curve with respect to ground (AUCg) and diurnal cortisol slope. Anxiety and caregiver burden were entered into linear regression models simultaneously.

**Results:**

While high levels of pre-surgical anxiety were positively associated with increased follow-up levels of AUCg (*β* = 0.30, *p* = 0.001), greater pre-surgery perceived burden score was associated with steeper cortisol slope (*β* = 0.27, *p* = 0.017) after controlling for a wide range of covariates.

**Conclusion:**

These outcomes support the utility of psychological interventions aimed to increase the awareness of caregiving tasks and demands in informal caregivers.

## Introduction

Informal caregiving for a sick relative or friend is a common experience in the UK, with estimates suggesting a 1-year prevalence of about 10% [1]. Caregiving for patients experiencing acute traumatic events such as coronary artery bypass graft (CABG) surgery often falls upon the spouse or partner of the patient. These caregivers have been found to experience disruption to their emotional and physical well-being. For example, elevated anxiety [2] and an increase in caregiver burden following the surgery [3] are notable problems in the caregiving experience. A review on the impact of an acute cardiac event on patients’ caregivers showed that caregivers are vulnerable to developing anxiety, depression, stress, caregiver burden and psychosocial and physical health problems [4]. Such burden has been associated with adverse physical health outcomes and premature mortality in caregivers [5]. The psychological and physical well-being of caregivers is not only important for their own health, but also for the effects on the patient’s psychological adjustment and management of illness [6].

One hypothesised mediator of the association between caregiver anxiety and burden and poor health outcomes is via activation of the neuroendocrine axis. Indeed, chronic stress, such as caregiving, has long been associated with cortisol dysregulation [7]. Chronic stress is hypothesised to lead to a state of allostasis, the wear and tear on the body’s stress system resulting from chronic overactivity or inactivity of physiological systems, including the hypothalamic-pituitary-adrenal (HPA) axis and resulting neuroendocrine dysregulation. Traditional caregiver studies have tended to look at caregivers of dementia patients in order to lend support to allostatic load theory [8–10]. This literature has expanded across the past decade to include research into non-elderly populations [11]. Moreover, other work has also studied the physiological consequences of caregiving for cancer patients [12]. However, whether the same pattern of findings is replicable in an acute caregiving situation, such as after CABG surgery, is currently less well understood.

Dysregulated cortisol has been associated with an array of poor health outcomes including an increased risk of all-cause and cardiovascular mortality in those with flatter diurnal slopes [13] and an increased risk of breast and lung cancer mortality again in those with flatter diurnal slopes [14, 15]. Other aspects of the diurnal profile of cortisol have also been associated with deleterious effects for health. For example, greater cortisol output across the day has been associated with the extent of coronary atherosclerosis [16]. Therefore, there is a need for studies which attempt to better understand the causes of cortisol dysregulation in ‘at risk’ groups such as caregivers.

The aim of the current study was to investigate the prospective relationship between two markers of pre-surgical distress, namely anxiety and perceived caregiver burden, and cortisol output across the day measured 2 months after surgery in partners of CABG patients. Moreover, a secondary aim was to examine the independent effects of the two caregiver burden subscales: difficulty and time, on cortisol measured 2 months after surgery.

## Method

### Participants

Participants were the caregivers (spouse or co-habiting partner) of patients who took part in the ARCS (Adjustment and Recovery after Cardiac Surgery) study; full details are published elsewhere [17]. Of 142 recruited participants, 39 had missing data on covariates leaving an analytic sample of 103 caregivers of patients undergoing elective first-time CABG surgery at St George’s Hospital, London; the 39 excluded caregivers did not differ from the analytic sample on any clinical or demographic variables. Cortisol area under the curve with respect to ground (AUCg) analyses had missing data on a small number of cases (*n* = 9), so separate *N*s are reported for these analyses. Participants were included if they were over 18 years old, were fluent in English, and if they were the primary caregiver. On average, participants completed baseline assessments 28.86 days before patients’ surgery and post-surgery assessment 60.94 days after the spouses’ surgery. All procedures were carried out with written informed consent of the participant. The study was approved by the South West London research ethics committee.

### Measures

#### Independent Variables: Anxiety and Caregiver Burden

Anxiety was assessed with the 7-items anxiety subscale of the Hospital Anxiety and Depression Scale (HADS) [18]. Participants were asked to rate the extent to which they had experienced each item (e.g. ‘worrying thoughts go through my mind’) in the previous 2 weeks. This scale has been shown to be a reliable measure of anxiety screening in the general population [19] with higher scores indicating greater anxiety (ranging from 0 to 21). The HADS questionnaire has previously been administered in studies of CABG caregivers [2] and in caregivers of patients undergoing cardiac surgery [20]. This subscale was used as a continuous variable (Cronbach’s alpha = 0.88).

Caregiver burden was measured using the 15-item Oberst Burden Scale (OCBS) [21], in which each caregiving task (e.g. emotional support, ‘being there’ for your partners) was evaluated with a 5-point Likert scale across two dimensions: difficulty and time. Each item is answered with reference to the question ‘How much time do you spend and how difficult is each activity for you to do?’ This scale was selected because it has been used almost exclusively in partners of patients undergoing CABG surgery [3, 22, 23]. It is considered suitable for use in cardiac patients’ partners as it assesses the burden related to everyday activities, which are necessary for the self-management of cardiac illness, covering a wide range of practical and personal care tasks*.* The total burden score was obtained by calculating the square root of the product of the two subscales (Cronbach’s alpha = 0.93). Both subscales and total score could range from 15 to 75, with higher scores indicating greater burden. In further analyses to look at the burden change between baseline and follow-up, the total burden score was split at the 75th percentile (23.87) to create low and high burden subgroups. For illustration purposes, we also divided caregiving difficulty in the same manner (75th percentile = 17.00) to create low and high caregiving difficulty groups.

#### Dependent Variables: Cortisol AUCg and Cortisol Slope

All details of the saliva collection protocol are provided elsewhere [17]. Briefly, participants’ pre- and post-surgery saliva samples were collected at 7 time points over the course of a single day (on waking, 30 min after awakening, 10 am, noon, 4 pm, 8 pm and bedtime) using Salivettes (Sarstedt, UK). On average, the samples were obtained 28.86 days before and 60.94 days after patients’ surgery; tubes were returned by post and were stored at − 20 °C for batch analysis at a later date by the University of Dresden (Germany) using a time-resolved immunoassay with fluorescence detection. Cortisol AUCg was derived from the trapezoid formula [24], with higher values indicating greater cortisol output across the day. Cortisol slope over the day was calculated as the reduction of cortisol per hour (nmol/L/h) using regression on cortisol values, excluding the 30 min after wakening sample [25]. Slope values closer to zero reflect flatter diurnal rhythms.

#### Covariates

Caregivers’ age, gender, number of people in the household (including the participant), occupation and smoking were self-reported at baseline, while patients’ clinical risk was assessed using the EuroSCORE (European System for Cardiac Operative Risk Evaluation) index [26]. Items were scored using the ‘logistic EuroSCORE’ method to generate a percentage mortality risk estimate. Caregivers listed their current occupation, which was classified according to the Office of National Statistics Standard Occupation Classification (SOC) 2010 index [27]. Participants were subsequently categorised into 9 groups, from high to low occupation, using SOC as an indicator of socioeconomic status (SES). Smoking was measured as a binary variable (smoker/non-smoker).

### Statistical Analysis

All outliers greater than 3 standard deviations (SD) from the mean were removed from the cortisol data. Cortisol data were normally distributed, so the raw scores are reported here. Change over time of cortisol AUCg and slope were assessed using paired *t* tests, which was also used to compare the change of burden from baseline to follow-up in those who at baseline were either in the high or low total burden group. Multiple linear regression was used to model the association between pre-surgery anxiety and caregiver burden and cortisol AUCg and slope values. Anxiety and caregiver burden were entered into models simultaneously in order to assess their independent effects. Similar findings were found entering caregiver burden and anxiety symptoms separately into models and so are not reported here. In both models, age, sex, SES, number in the household, smoking, patients’ EuroSCORE and baseline of the cortisol-dependent variable were entered as covariates. Given the large proportion of women in our study, secondary analyses were performed by entering mean-centred interaction terms into the models to examine the sex interaction with anxiety and caregiver burden. In further analyses, baseline caregiver burden was replaced by baseline caregiving difficulty and time subscales in fully adjusted regression models. Variance inflation factor (VIF) values and tolerance values were generated for all regression models to assess multicollinearity and the assumption was non violated (VIF < 10 and tolerance > 0.1). All data analyses were conducted using IBM SPSS Statistics version 22 (SPSS Inc.).

## Results

Demographic characteristics for all participants who completed baseline and follow-up are presented in Table 1. On average, anxiety levels significantly decreased over time (baseline: mean 6.39, SD 4.37; follow-up: mean 5.27, SD 3.26; *t* = 3.12, *p* = 0.002), while caregiver burden scores had a significant increase following the surgery (baseline: mean 21.66, SD 5.90; follow-up: mean 26.21, SD 7.76; *t* = − 6.89, *p* < 0.001). When we split the file by low/high baseline total burden, the low total burden group had a significant rise in burden from baseline to follow-up (*t* = −8.20, *p* < 0.001), while the high total burden group did not (*t* = −1.13, *p* = 0.269). Anxiety and caregiver burden at pre-surgical baseline were moderately correlated (*r* = 0.40, *p* < 0.001).

**Table 1:** Demographic, biological and psychological characteristics of the sample (*N* = 103)

Characteristic	Mean ± SD or *N* (%)
Baseline demographic variables	
Age	66.15 ± 8.31
Sex – Female	99 (96.1)
Occupation classification	
High	38(36.9)
Intermediate	39 (37.9)
Low	26 (25.2)
Household of two persons	92 (89.3)
Smoker	3 (2.9)
Cortisol variables	
Cortisol AUC_g_^a^–baseline (nmol/L)*	7853.56 ± 2531.22
Cortisol AUC_g_^a^–follow-up (nmol/L)*	7479.31 ± 2407.91
Cortisol slope–baseline (nmol/L/h)	0.016 ± 0.010
Cortisol slope–follow-up (nmol/L/h)	0.018 ± 0.009
Baseline psychological distress	
Anxiety^b^	6.39 ± 4.37
Total caregiver burden^c^	21.66 ± 5.90
Caregiving time^c^	27.25 ± 8.90
Caregiving difficulty^c^	17.40 ± 4.76
Patients’ clinical variable	
EuroSCORE (%)^d^	4.25 ± 3.55

^*^N = 94

^a^Cortisol area under the curve with respect to the ground

^b^Anxiety subscale of HADS (Hospital Anxiety Depression Scale)

^c^OBS (Oberst Burden Scale)

^d^European System for Cardiac Operative Risk Evaluation

Caregivers’ cortisol output over the day (AUCg) were higher before patients’ surgery compared with follow-up (*t* = 1.46, *p* = 0.148), while baseline cortisol slope was slightly flatter compared with post-surgery slope values (*t* = −1.41, *p* = 0.163). Nevertheless, these changes were non-significant.

Regression analyses showed that caregiver anxiety levels at pre-surgery baseline were associated with cortisol AUCg following surgery (*β* = 0.29, *p* = 0.001), while caregiver burden did not (*β* = − 0.02, *p* = 0.786), after controlling for all covariates. Baseline cortisol AUCg (*β* = 0.49, *p* < 0.001) and gender (*β* = 0.20, *p* = 0.018) were also significantly positively associated with cortisol AUCg cortisol post-surgery follow-up. The model accounted for 44% of variance in cortisol AUCg. See Table 2 for these results.

**Table 2:** Multiple regression on baseline caregiver psychological distress predicting cortisol AUCg (*N* = 94)

Model	*B*	SE	95% CI	*β*	*p*
Step 1					
Total caregiver burden^a^	23.176	43.455	[− 63.143; 109.495]	0.057	0.595
Anxiety^b^	160.433	60.598	[40.063; 280.802]	0.282	0.010
Step 2					
Total caregiver burden^a^	− 10.252	37.575	[− 84.973; 64.470]	− 0.025	0.786
Anxiety^b^	167.074	50.497	[66.655; 267.494]	0.294	0.001
Age	− 11.856	33.555	[− 78.585; 54.872]	− 0.038	0.725
Gender	2762.889	1145.771	[484.398; 5041.379]	0.203	0.018
Number in household	553.923	408.506	[− 258.436; 1366.282]	0.126	0.179
Occupation	− 141.418	106.912	[− 354.024; 71.188]	− 0.112	0.190
Smoking	− 2082.981	1192.525	[− 4454.447; 288.485]	− 0.153	0.084
Patients’ EuroSCORE^c^	92.978	62.721	[− 31.751; 217.706]	0.142	0.142
Baseline cortisol AUCg^d^	0.471	0.086	[0.300; 0.642]	0.496	< 0.001

^a^OBS (Oberst Burden Scale)

^b^Anxiety subscale of HADS (Hospital Anxiety Depression Scale)

^c^European System for Cardiac Operative Risk Evaluation

^d^Cortisol area under the curve with respect to the ground

In a separate model (See Table 3), after controlling for covariates, pre-surgery caregiver burden was significantly associated with cortisol slope post-surgery (*β* = 0.27, *p* = 0.017), but baseline anxiety was not (*β* = − 0.05, *p* = 0.630). Greater pre-surgical burden was associated with steeper cortisol decline over the day. The baseline values of cortisol slope were positively associated with cortisol slope at follow-up (*β* = 0.28, *p* = 0.008). There were no other significant variables. The model accounted for 14% of variance in cortisol slope.

**Table 3:** Multiple regression on baseline caregiver psychological distress predicting cortisol slope (*N* = 103)

Model	*B*	SE	95% CI	*β*	*p*
Step 1					
Total caregiver burden^a^	0.0004	0.0002	[0.00007, 0.001]	0.258	0.017
Anxiety^b^	− 0.0002	0.0002	[− 0.001; 0.0002]	− 0.103	0.334
Step 2					
Total caregiver burden^a^	0.0004	0.0002	[0.0001; 0.001]	0.272	0.017
Anxiety^b^	− 0.0001	0.0002	[− 0.001; 0.0003]	− 0.052	0.630
Age	− 0.00003	0.0001	[− 0.0003; 0.0002]	− 0.029	0.821
Gender	0.0001	0.005	[− 0.009; 0.010]	0.002	0.982
Number in household	0.002	0.002	[− 0.001; 0.006]	0.131	0.249
Occupation	0.0004	0.0005	[− 0.001; 0.001]	− 0.080	0.427
Smoking	0.002	0.005	[− 0.009; 0.013]	0.040	0.689
Patients’ EuroSCORE^c^	0.0001	0.0003	[− 0.0005; 0.001]	0.038	0.735
Baseline cortisol slope	0.250	0.092	[0.068; 0.433]	0.281	0.008

^a^OBS (Oberst Burden Scale)

^b^Anxiety subscale of HADS (Hospital Anxiety Depression Scale)

^c^European System for Cardiac Operative Risk Evaluation

In secondary analyses, we examined the sex × anxiety interaction by entering the mean-centred interaction term in models to predict cortisol AUCg. There was no significant interaction (*p* = 0.983). We also examined the sex × burden interaction by entering the mean-centred interaction term in models to predict cortisol slope. Again, there was no significant interaction (*p* = 0.208).

**Fig. 1:**
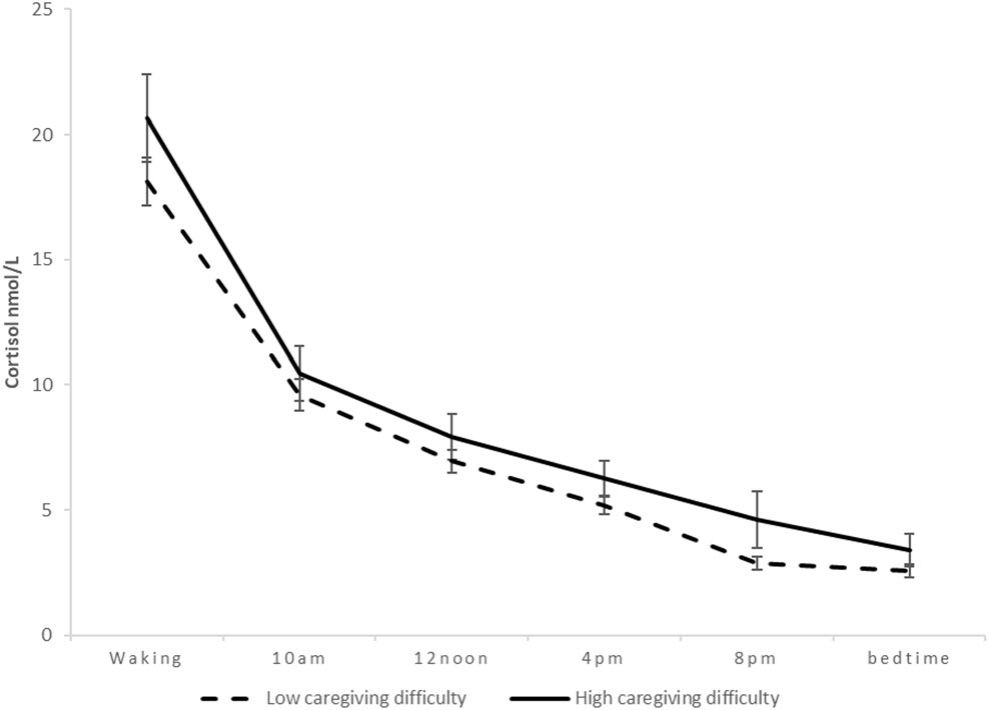
Diurnal cortisol output of caregivers 60.94 days following CABG surgery by baseline levels of caregiving difficulty. Bars indicate standard errors of the mean (*N* = 103)

In further analyses using the caregiver burden subscales (caregiving time and caregiving difficulty), pre-surgical caregiving difficulty (*β* = 0.328, *p* = 0.016), but not time (*β* = −0.020, *p* = 0.877), was associated with cortisol slope post-surgery. The relationship between post-surgery cortisol slope and baseline caregiving difficulty values is illustrated in Fig. 1.

## Discussion

We found that higher pre-surgical anxiety was associated with greater post-surgery cortisol AUCg and that increased caregiver burden at baseline was associated with a steeper cortisol slope at follow-up. These associations were independent of all covariates included in the models and of the other distress marker, since anxiety and caregiver burden were entered simultaneously into regression models. These are the first findings to our knowledge that have demonstrated the effects of pre-surgery distress on post-surgery cortisol response in caregivers of CABG patients, suggesting the importance of providing support to partners who take on acute caregiving roles.

In the first prospective model, we found that greater anxiety, but not burden, prior to the patient’s surgery was associated with greater post-surgery cortisol AUCg. These findings lend support to allostatic load theory in caregivers [8] and are further supported by previous research which has found an effect of anxiety on greater cortisol AUC in cross-sectional analyses [28]. Our study was able to explore longitudinal effects, suggesting pre-surgical anxiety may have a detrimental effect on caregivers’ cortisol regulation up to 2 months after the CABG surgery.

Perhaps less intuitively we also found that greater pre-surgical caregiving burden, but not anxiety, was associated with a steeper slope at the post-surgery follow-up. A steeper slope is usually considered to be adaptive [29], so our finding is in opposition to what we might expect based on allostatic load theory. However, we propose that this finding can be explained by the caregiving situation whereby the baseline measurement of caregiver burden indicates a readiness to provide care following patient’s surgery. In this scenario, higher pre-surgical caregiver burden indicates a greater aptitude to meet the additional caregiving requirements following surgery. Support for this hypothesis is lent by the fact we observed different increases in burden over time when we split our file by baseline total burden. Caregivers who had a high level of burden at baseline did not show a significant increase in burden at follow-up, while the low baseline burden group did. Previous findings have shown that increased anticipatory stress before a psychosocial stressor predicted a stepper cortisol recovery following a laboratory stress test [30]. These authors explained this result as a possible healthy effect of anticipatory stress inducing high vigilance and a readiness to respond to possible subsequent stressors. However, this hypothesis requires further testing in future studies.

A meta-analysis of psychosocial interventions for patients and their informal caregivers showed overall positive effects for outcomes including depression and anxiety [31]. However, there is a lack of research investigating the effects of interventions in caregivers of acutely ill patients such as those in our CABG surgery sample. This has been attributed to reasons such as a lack of resources, interest and knowledge among staff, and practical and psychological barriers among patients and partners [32]. Spouses and partners of CABG surgery patients are often faced with uncertainty about the responsibilities of the caregiving role following the patients’ discharge which contribute to anxiety, burden and worries about their preparedness for responding properly to patient demands [33]. A systematic review has revealed that a modest number of intervention studies (*N* = 7) targeting coronary heart disease caregivers’ well-being have shown trends toward improvements in anxiety, knowledge and satisfaction in care [34]. The VITAL telehealth programme used a randomised controlled trial design to investigate the effects of a telephone intervention on CABG patients and their caregiving partners’ psychological state in the period following hospital discharge. This study demonstrated a positive effect of the intervention on anxiety and depression symptoms [35]. Such work indicates the potential benefits of intervening with this population, although further research is needed to examine the long-term effects of such trials.

Our study has a number of strengths. First, we used a longitudinal design so were able to demonstrate a prospective relationship over time. Moreover, cortisol was measured at different time points across the day allowing us to get a reliable cortisol profile. However, limitations include our reliance on questionnaire measures to assess anxiety and burden symptoms, which restricts us from generalising our results to clinical samples. Furthermore, our sample was composed mainly of female participants. This prevalence is explained by the predominance of men in the UK CABG surgical population [36]; however, our findings may not be readily generalisable to male caregivers. Lastly, both pre- and post-surgery cortisol was assessed only on a single day.

In conclusion, greater anxiety in caregivers 1 month before patients’ surgery was associated with higher cortisol output across the day (AUCg) at 2-months follow-up, while increased pre-surgical level of caregiver burden was significantly associated with a steeper cortisol slope following patients’ surgery. More research is needed to test the types of interventions that would be most acceptable and efficacious in this kind of caregivers.
